# Synthesis and spectroscopic and structural characterization of three new 2-methyl-4-styryl­quinolines formed using Friedländer reactions between (2-amino­phen­yl)chalcones and acetone

**DOI:** 10.1107/S2053229622008634

**Published:** 2022-09-05

**Authors:** Diana Rocío Vera, Juan P. Mantilla, Alirio Palma, Justo Cobo, Christopher Glidewell

**Affiliations:** a Laboratorio de Síntesis Orgánica, Escuela de Química, Universidad Industrial de Santander, AA 678, Bucaramanga, Colombia; b Departamento de Química Inorgánica y Orgánica, Universidad de Jaén, 23071 Jaén, Spain; c School of Chemistry, University of St Andrews, St Andrews, Fife KY16 9ST, United Kingdom; University of Strathclyde, United Kingdom

**Keywords:** synthesis, quinoline, Friedlander reaction, NMR spectroscopy, crystal structure, mol­ecular conformation, hydrogen bonding, supra­molecular assembly

## Abstract

Three new 2-methyl-4-styryl­quinoline derivatives have been synthesized in high yields using Friedländer reactions between chalcones [1-(2-amino­phen­yl)-3-aryl­prop-2-en-1-ones] and acetone, and characterized using IR, ^1^H and ^13^C NMR spectroscopy, and mass spectrometry, and by crystal structure analysis. In (*E*)-4-(4-fluoro­styr­yl)-2-methyl­quinoline, C_18_H_14_FN, (I), the mol­ecules are joined into cyclic centrosymmetric dimers by C—H⋯N hydrogen bonds and these dimers are linked into sheets by π–π stacking inter­actions. The mol­ecules of (*E*)-2-methyl-4-[4-(tri­fluoro­meth­yl)styr­yl]quinoline, C_19_H_14_F_3_N, (II), are linked into cyclic centrosymmetric dimers by C—H⋯π hydrogen bonds and these dimers are linked into chains by a single π–π stacking inter­action. There are no significant hydrogen bonds in the structure of (*E*)-4-(2,6-di­chloro­styr­yl)-2-methyl­quinoline, C_18_H_13_Cl_2_N, (III), but mol­ecules related by translation along [010] form stacks with an inter­molecular spacing of only 3.8628 (2) Å. Comparisons are made with the structures of some related com­pounds.

## Abstract 2

The syntheses and structures are reported for three 4-styryl­quinoline derivatives formed by reactions between (2-amino­phen­yl)chalcones and acetone.

## Introduction

The quinoline nucleus constitutes a privileged scaffold because of the wide spectrum of promising biological activity exhibited by its derivatives (Kumar *et al.*, 2009 ▸). Among quinoline derivatives, 2-styryl­quinolines have been studied extensively, mainly because of their potential as inhibitors of HIV-1 integrase (Leonard & Roy, 2008 ▸; Mahajan *et al.*, 2018 ▸; Mousnier *et al.*, 2004 ▸) and as anti­microbial (Kamal *et al.*, 2015 ▸), anti­fungal (Cieslik *et al.*, 2012 ▸) and anti­cancer agents (Mrozek-Wilczkiewicz *et al.*, 2015 ▸, 2019 ▸).

Accordingly, considerable efforts have been made in the development of effective methods for accessing new com­pounds containing the styryl­quinoline scaffold (Musiol, 2020 ▸). Unlike 2-styryl­quinolines, the 4-styryl­quinoline regioisomers have been studied much less, with few published reports related to their synthesis and biological evaluation, which is probably due, at least in part, to a lack of generally applicable methodologies for their synthesis. In general, the published syntheses of 4-styryl­quinolines have involved Heck coupling between 4-halo­quinolines and different ar­yl–vinyl com­pounds (Omar & Hormi, 2009 ▸), and Knoevenagel-type condensation reactions between 4-methyl­quinolines and aromatic aldehydes using expensive and toxic heavy-metal catalysts (Jamal *et al.*, 2016 ▸) or microwave irradiation (Lee *et al.*, 2009 ▸). The use of palladium catalysts in the cross-coupling reaction between 4-chloro­quinolines and alkenyltri­fluoro­borates under harsh reaction conditions has also been reported (Alacid & Nájera, 2009 ▸). Nonetheless, there still remains a need for alternative approaches for the construction of 4-styryl­quinolines starting from readily accessible materials and characterized by high atom efficiency and low cost.

In this context, and as part of an ongoing program exploring the rational use of synthetically available 1-(2-amino­phen­yl)-3-aryl­prop-2-en-1-ones (Meléndez *et al.*, 2020 ▸) as appropriate precursors for the synthesis of novel quinoline derivatives, we have recently described a simple and efficient one-pot synthetic approach based on the Friedländer reaction to obtain polysubstituted 2-methyl-4-styryl­quinolines starting from these simple precursors and different 1,3-dicarbonyl com­pounds (Meléndez *et al.*, 2020 ▸).

**Figure 1:**
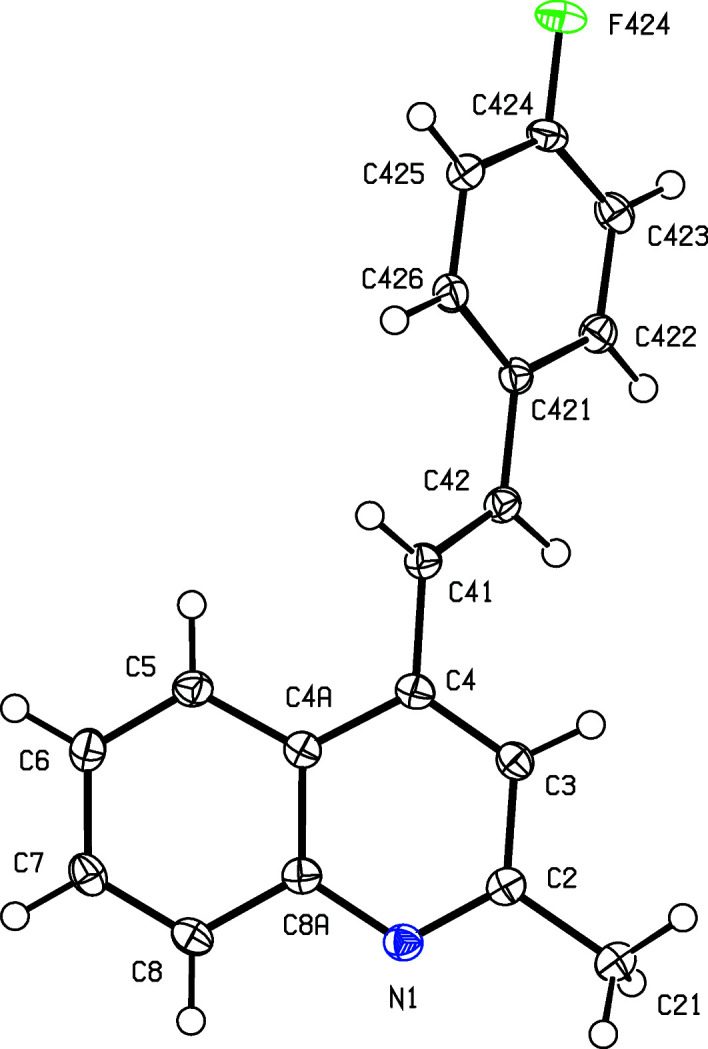
The mol­ecular structure of com­pound (I), showing the atom-labelling scheme. Displacement ellipsoids are drawn at the 50% probability level.

To expand further both the synthetic utility of 1-(2-amino­phen­yl)-3-aryl­prop-2-en-1-ones and the flexibility of our ap­proach, we report here the synthesis, characterization and mol­ecular and supra­molecular structures of a matched set of three closely-related quinoline derivatives, namely, (*E*)-4-(4-fluoro­styr­yl)-2-methyl­quinoline, (I), (*E*)-2-methyl-4-[4-(tri­fluoro­meth­yl)styr­yl]quinoline, (II), and (*E*)-4-(2,6-di­chloro­styr­yl)-2-methyl­quinoline, (III) (Scheme 1 and Figs. 1 ▸–3 ▸ ▸), which differ only in the nature of the substituents at the C4 and C2/C6 positions on the benzene ring of the styryl fragment. Using our synthetic approach (Meléndez *et al.*, 2020 ▸), (*E*)-1-(2-amino­phen­yl)-3-aryl­prop-2-en-1-ones of type (*A*) (Scheme 1) were subjected to Friedlander annulation with an excess of acetone in glacial acetic acid at 373 K, to provide the products (I)–(III) with yields in the range 77–94% (Scheme 1). These new 2-methyl­quinoline derivatives are intended for use as key pre­cursors in the further development of more com­plex mol­ecules of possible biological value, such as the bis-styryl­quinolines (IV) (Scheme 2), (4-styrylquinolin-2-yl)chalcones of the type (V) and the mol­ecular hybrids of types (VI) and (VII). 
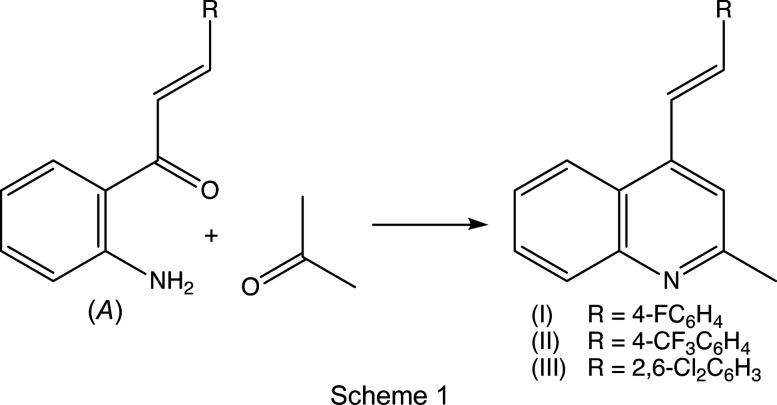


## Experimental

### Synthesis and crystallization

For the synthesis of com­pounds (I)–(III), a mixture of the appropriate 1-(2-amino­phen­yl)-3-aryl­prop-2-en-1-ones (*A*) (Meléndez *et al.*, 2020 ▸; see Scheme 1) (1.0 mmol) and acetone (12.0 mmol) in glacial acetic acid (3 ml) was stirred magnetically and heated at 353 K until the reactions were com­plete, as judged by the com­plete consumption of (*A*) (as monitored by thin-layer chromatography, TLC); the reaction times for com­pletion were 15 h for (I), 19 h for (II) and 14 h for (III). Each reaction mixture was then neutralized with a saturated aqueous sodium carbonate solution and extracted with ethyl acetate (3 × 50 ml). The combined organic layers were washed with water and dried over anhydrous sodium sulfate, and the solvent was then removed under reduced pressure. In each case, the resulting crude product was purified by flash chromatography on silica-gel using hexa­ne–ethyl acetate mixtures as eluent (com­positions ranged from 7:1 to 2:1 *v*/*v*) to give the required solid com­pounds (I)–(III). Crystallization from hexa­ne–ethyl acetate (10:1 *v*/*v*) at ambient temperature and in the presence of air gave crystals suitable for single-crystal X-ray diffraction; these were yellow for (I) and (III), and colourless for (II). 
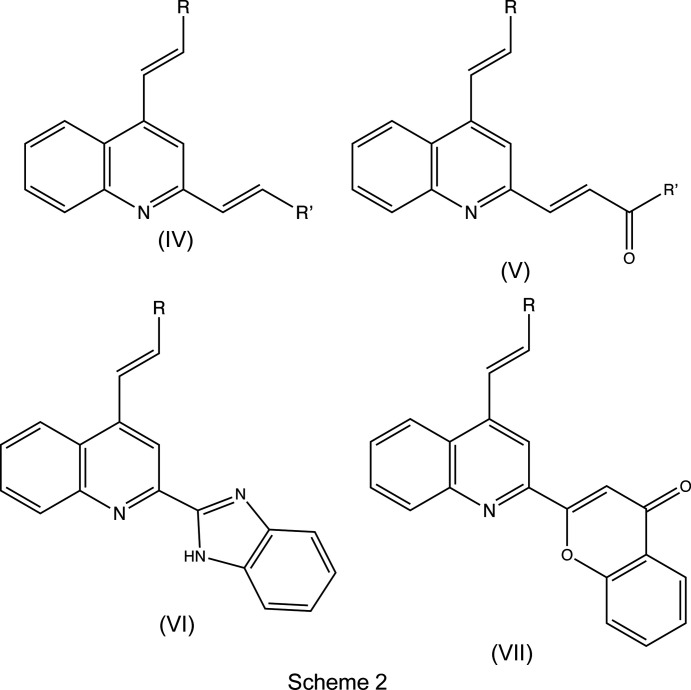


Compound (I): yield 0.21g (84%), m.p. 395–397 K, *R*_f_ = 0.28 (16.6% ethyl acetate–hexa­ne). FT–IR (ATR, cm^−1^): 1632 (C=N), 1598 (C=C_vin­yl_), 1587 (C=C_arom_), 1506 (C=C_arom_), 965 (=C—H_*trans*_). NMR (CDCl_3_): δ(^1^H) 8.13 (*dd*, *J* = 8.4, 1.4 Hz, 1H, H5), 8.05 (*dd*, *J* = 8.4, 1.6 Hz, 1H, H8), 7.69 (*ddd*, *J* = 8.4, 6.9, 1.4 Hz, 1H, H7), 7.68 (*d*, *J* = 16.1 Hz, 1H, H_A_—C=), 7.56–7.61 (*m*, 2H, H2′, H6′), 7.52 (*ddd*, *J* = 8.3, 6.9, 1.4 Hz, 1H, H6), 7.47 (*s*, 1H, H3), 7.27 (*d*, *J* = 16.1 Hz, 1H, =CH_B_), 7.09–7.14 (*m*, 2H, H3′, H5′), 2.77 (*s*, 3H, 2-CH_3_); δ(^13^C) 163.0 (*d*, *J* = 248.9 Hz, C4′), 158.7 (C2), 148.4 (C8a), 142.8 (C4), 133.6 (=CH_B_), 132.9 (*d*, *J* = 3.6 Hz, C1′), 129.4 (C8), 129.3 (C7), 128.7 (*d*, *J* = 8.1 Hz, C2′, C6′), 125.7 (C6), 124.7 (C4a), 123.2 (C5), 122.9 (*d*, *J* = 2.3 Hz, H_A_C=), 117.9 (C3), 115.9 (*d*, *J* = 21.9 Hz, C3′, C5′), 25.4 (2-CH_3_). HRMS (ESI^+^) *m*/*z* found for [*M* + H]^+^ 264.1181, C_18_H_14_FN requires 263.11

Compound (II): yield (77%); m.p. 391–392 K, *R*_f_ = 0.34 (50% ethyl acetate–hexa­ne). FT–IR (ATR, cm^−1^): 1620 (C=N), 1587 (C=C_vin­yl_), 1505 (C=C_arom_), 1408 (C=C_arom_), 964 (=C—H_*trans*_). NMR (CDCl_3_): δ(^1^H) 8.13 (*dd*, *J* = 8.3, 1.4 Hz, 1H, H5), 8.06 (*dd*, *J* = 8.4, 1.5, Hz, 1H, H8), 7.86 (*d*, *J* = 16.1 Hz, 1H, H_A_—C=), 7.72 (*ddd*, *J* = 8.3, 6.9, 1.4 Hz, 1H, H7), 7.72 (*d*, *J* = 8.5 Hz, 2H, H2′, H6′), 7.68 (*d*, *J* = 8.5 Hz, 2H, H3′, H5′), 7.54 (*ddd*, *J* = 8.3, 6.8, 1.3 Hz, 1H, H6), 7.50 (*d*, *J* = 0.7 Hz, 1H, H3), 7.32 (*d*, *J* = 16.1 Hz, 1H, =CH_B_), 2.79 (*s*, 3H, 2-CH_3_); δ (^13^C) 158.8 (C2), 148.5 (C8a), 142.3 (C4), 140.0 (C1′), 133.2 (=CH_B_), 130.3 (*d*, *J* = 32.4 Hz, C4′), 129.5 (C7), 129.4 (C8), 127.2 (C2′, C6′, C3′, C5′), 125.9 (*q*, *J* = 3.7 Hz, 4-CF_3_), 125.4 (C6), 124.7 (C4a), 123.1 (C5), 122.7 (H_A_—C=), 118.2 (C3), 25.4 (2-CH_3_). HRMS (ESI^+^) *m*/*z* found for [*M* + H]^+^ 314.115, C_19_H_14_F_3_N requires 313.1078.

Compound (III): yield 0.25 g (94%), m.p. 410-412 K, *R*_f_ = 0.31 (12.5% ethyl acetate–hexa­ne). FT–IR (ATR, cm^−1^): 1629 (C=N), 1593 (C=C_vin­yl_), 1554 (C=C_arom_), 1505 (C=C_arom_), 959 (=C—H_*trans*_). NMR (CDCl_3_): δ(^1^H) 8.10 (*dd*, *J* = 8.5, 1.4 Hz, 1H, H5), 8.06 (*dd*, *J* = 8.5, 1.4 Hz, 1H, H8), 7.85 (*dd*, *J* = 16.5, 0.87 Hz, 1H, H_A_—C=), 7.70 (*ddd*, *J* = 8.4, 6.9, 1.4 Hz, 1H, H7), 7.53 (*ddd*, *J* = 8.4, 6.9, 1.3 Hz, 1H, H6), 7.53 (*s*, 1H, H3), 7.41 (*d*, *J* = 8.0 Hz, 2H, H3′, H5′), 7.18 (*dd*, *J* = 8.4, 7.7 Hz, 1H, H4′), 7.28 (*d*, *J* = 16.5 Hz, 1H, =CH_B_), 2.80 (*s*, 3H, 2-CH_3_). δ (^13^C) 158.8 (C2), 148.4 (C8a), 142.2 (C4), 137.2 (C1′), 134.7 (C2′, C6′), 133.8 (C3′), 132.4 (H_A_C=), 130.9 (=CH_B_), 130.1 (C4′), 129.3 (C7), 129.4 (C8), 127.4 (C5′), 125.9 (C6), 124.8 (C4a), 123.6 (C5), 118.5 (C3), 25.5 (2-CH_3_). HRMS (ESI^+^) *m*/*z* found for [*M* + H]^+^ 314.0500, C_18_H_13_Cl_2_N requires 313.0425.

### Refinement

Crystal data, data collection and refinement details are summarized in Table 1 ▸. A small number of bad outlier reflections [ 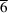 36 for (I), 204 and 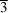 36 for (II), and 16,0,0 and 339 for (III)] were omitted from the data sets. All H atoms were located in difference maps and then treated as riding atoms in geometrically idealized positions, with C—H distances of 0.95 (alkenic and aromatic) and 0.98 Å (CH_3_), and with *U*_iso_(H) = *kU*_eq_(C), where *k* = 1.5 for the methyl groups, which were permitted to rotate but not to tilt, and 1.2 for all other H atoms.

## Results and discussion

All com­pounds were fully characterized by standard spectroscopic and analytical methods. In the IR spectra of (I)–(III), the absence of any N—H stretching bands around 3275–3285 cm^−1^, which are characteristic in the spectra of (2-am­ino­phen­yl)chalcone precurors, was used for monitoring the formation of the quinoline ring. The formation of the 4-styryl­quinoline scaffold was confirmed by a detailed analysis of the ^1^H, ^13^C and 2D NMR spectra, which showed no signals arising from the H atoms of the amino group; neither were there any signals from the carbonyl groups which had been present in the precursor chalcones. Instead, the ^13^C spectra of the products contained signals from a new C_ar­yl_—H unit (C-3) in the range δ 117.9–118.5, and two new quaternary aromatic C atoms at δ 158.7–158.8 (C-2) and 142.2–142.8 (C-4). As in the spectra of the precursor chalcones, the ^1^H spectra of products (I)–(III) contained signals from the *trans* vinylic protons –CH_A_=CH_B_–, appearing as two doublets (see Section 2.1). Finally, definitive confirmation of the mol­ecular constitutions and the regio- and stereochemistry for com­pounds (I)–(III) was established by means of single-crystal X-ray diffraction, and thus we report here also the mol­ecular and supra­molecular structures for all three examples (Figs. 1 ▸–3 ▸ ▸).

These new 2-methyl­quinoline derivatives (I)–(III) are intended for use as key precursors in the further development of more com­plex mol­ecules of possible biological value, such as the bis-styryl­quinolines (IV) (Scheme 2), (4-styrylquinolin-2-yl)chalcones of the type (V), and the mol­ecular hybrids of types (VI) and (VII), and the work reported here can be regarded as a continuation of an earlier crystallographic study which reported the structures of 2-methyl-4-styryl­quinolines having either acetyl or carboeth­oxy functionalities at position C3 (Rodríguez *et al.*, 2020 ▸).

The mol­ecules of com­pounds (I)–(III) exhibit no inter­nal symmetry, as indicated by the key torsion angles (Table 2 ▸). They are thus not superimposable upon their mirror images and hence they are all conformationally chiral (Moss, 1996 ▸; Flack & Bernardinelli, 1999 ▸). The space groups (Table 1 ▸) confirm that the crystals of each com­pound contain equal numbers of the two conformational enanti­omers; for each com­pound, the reference mol­ecule was selected as one having a positive sign for the torsion angle C3—C4—C41—C42 (Table 2 ▸). Only in com­pound (II) is the styryl fragment involved in direction-specific inter­molecular inter­actions, as discussed below, and hence there appears to be no simple inter­pretation of the conformational differences in com­pounds (I)–(III), other than to note that the barriers to rotation about the C—C single bonds are generally quite low, typically a few kJ mol^−1^ (Alkorta & Elguero, 1998 ▸).

The supra­molecular assembly in com­pounds (I)–(III) is very simple (Table 3 ▸). There is a single hydrogen bond in the structure of (I). In the structure of (II), there is a C—H⋯π(arene) hydrogen bond, but for the inter­molecular C—H⋯N contact, the H⋯N distance exceeds the sum, 2.70 Å, of the van der Waals radii for these atoms (Rowland & Taylor, 1996 ▸); hence, this is just a normal inter­molecular contact with no associated attractive inter­action which could be regarded as structurally significant. The C—H⋯N contact in com­pound (III) involves a methyl group (Table 3 ▸), where the C—H bonds are of low acidity. More significantly, methyl groups are, in general, likely to be undergoing very fast rotation about the adjacent C—C bond in the solid state (Riddell & Rogerson, 1996 ▸, 1997 ▸). For methyl groups bonded to planar fragments such as aryl rings, the sixfold barrier to rotation is usually very small, only a few J mol^−1^ rather than the typical order of magnitude in kJ mol^−1^ (Naylor & Wilson, 1957 ▸; Tannenbaum *et al.*, 1956 ▸). Hence, this contact cannot be regarded as structurally significant. There are π–π stacking inter­actions in each structure.

**Figure 4:**
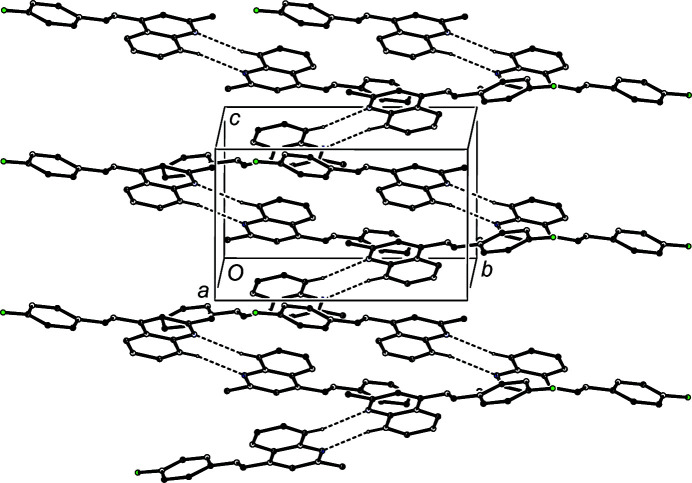
Part of the crystal structure of com­pound (I), showing the formation of a π-stacked sheet of hydrogen-bonded dimers lying parallel to (100). Hydrogen bonds are drawn as dashed lines and, for the sake of clarity, H atoms not involved in the motif shown have been omitted.

In the structure of (I), inversion-related pairs of mol­ecules are linked by almost linear C—H⋯N hydrogen bonds (Table 3 ▸) to form centrosymmetric dimers characterized by an 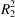 (8) motif (Etter, 1990 ▸; Etter *et al.*, 1990 ▸; Bernstein *et al.*, 1995 ▸) (Fig. 4 ▸). Dimers of this type are linked into sheets by π–π stacking inter­actions; the quinoline units of the mol­ecule at (*x*, *y*, *z*), makes dihedral angles of 9.21 (7)° with the corresponding rings of the mol­ecules at (*x*, −*y* + 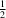 , *z* + 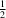 ) and (*x*, −*y* + 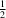 , *z* − 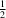 ), with ring-centroid separations of 3.7682 (9) Å in each case, with the shortest distance between the centroid of one ring and the plane of the other of 3.5610 (6) Å. The combination of inversion and glide-plane operations leads to the formation of a sheet of π-stacked dimers lying parallel to (100) (Fig. 4 ▸).

**Figure 5:**
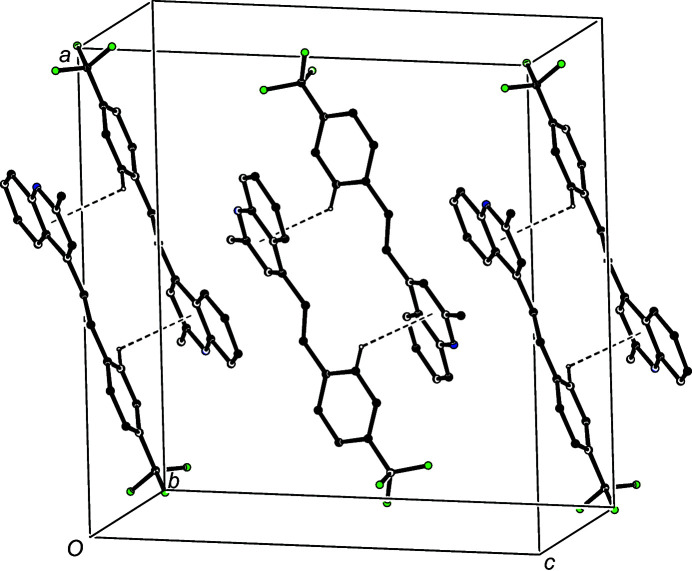
Part of the crystal structure of com­pound (II), showing the formation of a π-stacked chain of hydrogen-bonded dimers running parallel to [001] Hydrogen bonds are drawn as dashed lines and, for the sake of clarity, H atoms not involved in the motif shown have been omitted.

In the structure of com­pound (II), inversion-related pairs of mol­ecules are linked by a C—H⋯π(arene) hydrogen bond to form centrosymmetric dimers (Fig. 5 ▸), and these dimers are linked into chains by a single π–π stacking inter­action; the heterocyclic rings in the mol­ecules at (*x*, *y*, *z*) and (−*x* + 1, *y*, −*z* + 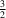 ) are strictly parallel, with an inter­planar spacing of 3.5058 (6) Å and a ring-centroid separation of 3.6845 (9) Å, corresponding to a ring-centroid offset of 1.1335 (12) Å. By this means, the hydrogen-bonded dimers are linked into a chain running parallel to [001] (Fig. 5 ▸).

**Figure 6:**
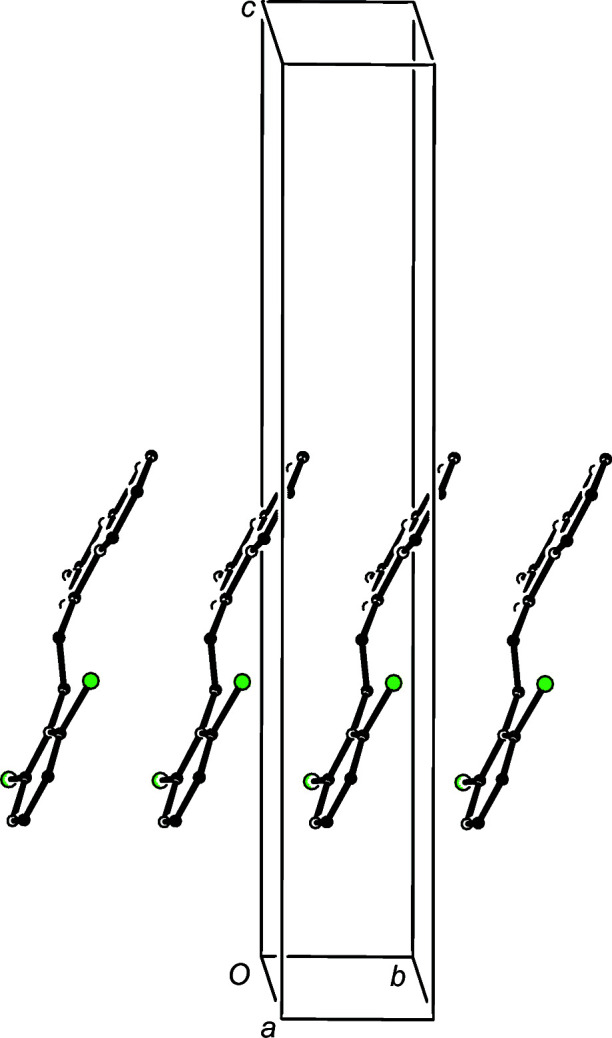
Part of the crystal structure of com­pound (III), showing the formation of a π-stacked chain of hydrogen-bonded dimers running parallel to [010]. Hydrogen bonds are drawn as dashed lines and, for the sake of clarity, H atoms not involved in the motif shown have been omitted.

**Figure 7:**
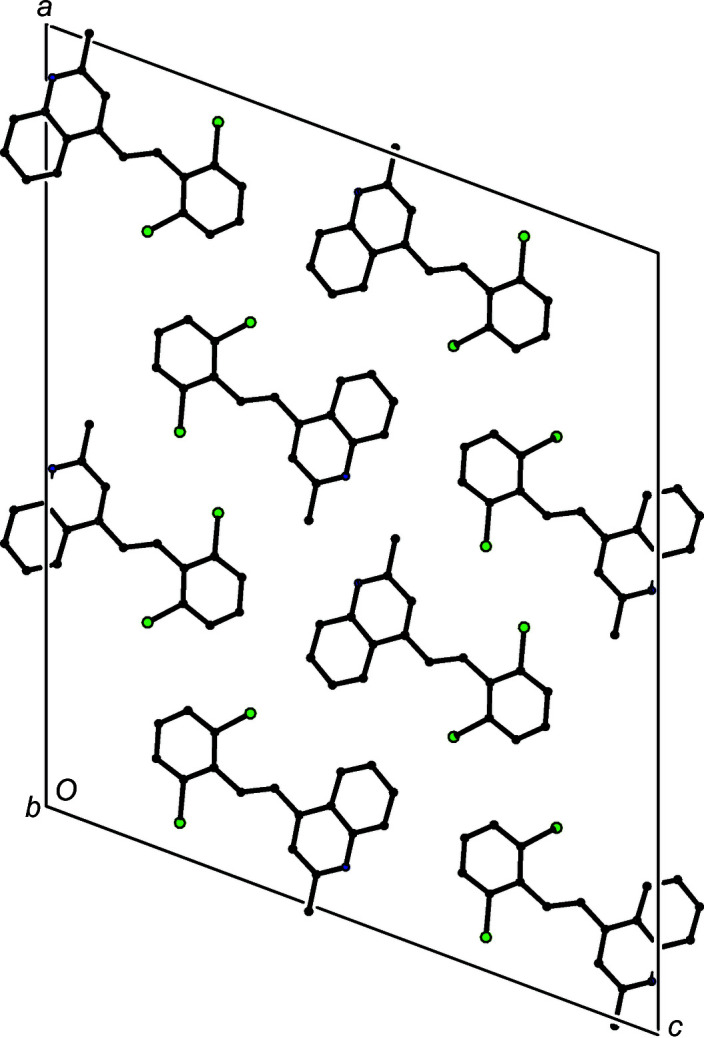
A projection along [010] of part of the crystal structure of com­pound (III), showing the arrangement of the mol­ecular stacks within the unit cell. For the sake of clarity, all H atoms have been omitted.

Although there are no hydrogen bonds in the structure of com­pound (III), the mol­ecules which are related by translation along the [010] direction are stacked precisely in register with a spacing equal to the unit-cell vector *b* = 3.8629 (2) Å (Fig. 6 ▸). Eight stacks of this kind pass through each unit cell (Fig. 7 ▸), but there are no direction-specific inter­actions between adjacent stacks.

We have previously reported (Rodríguez *et al.*, 2020 ▸) the synthesis and structures of a number of 4-styryl­quinoline derivatives carrying either acetyl or carboeth­oxy substituents at position C-3. Of these, three closely related acetyl derivatives were found to be isomorphous, with their mol­ecules linked into simple *C*(6) chains by a single C—H⋯O hydrogen bond. By contrast, the matching set of carboeth­oxy derivatives all exhibited different crystallization characteristics and different modes of supra­molecular assembly, with one forming *C*(13) chains and the other two forming cyclic centrosymmetric dimers involving C—H⋯O hydrogen bonds in one case and C—H⋯π hydrogen bonds in the other. In addition, two other examples carrying acyl substituents have been reported (Meléndez *et al.*, 2020 ▸) on a proof-of-structure basis without detailed structure analysis or description, but subsequent re-examination (Rodríguez *et al.*, 2020 ▸) found a com­plex sheet structure in one of them, but no significant inter­molecular inter­actions in the other.

The structures of a number of other styryl­quinolines are recorded in the Cambridge Structural Database (CSD; Groom *et al.*, 2016 ▸), but it is striking that the majority of these structures are of 2-styryl­quinoline derivatives, along with those of a small number of 8-styryl­quinolines. This may reflect, at least in part, a lack of efficient, straightforward and versatile routes to other isomeric styryl­quinolines. The structure of 2-styryl­quinoline itself has been reported three times (Valle *et al.*, 1986 ▸; Gulakova *et al.*, 2011 ▸; Kuz’mina *et al.*, 2011 ▸), as have those of 2-[2-(4-methyl­phen­yl)vin­yl]quinoline (Gulakova *et al.*, 2011 ▸; Kuz’mina *et al.*, 2011 ▸; Das *et al.*, 2019 ▸) and 2-[2-(3,4-meth­oxy­phen­yl)vin­yl]quinolone (Gulakova *et al.*, 2011 ▸; Kuz’mina *et al.*, 2011 ▸; Sharma *et al.*, 2021 ▸). There are two reports on the structure of 2-[2-(3-nitro­phen­yl)vin­yl]quinoline (Gulakova *et al.*, 2011 ▸; Kuz’mina *et al.*, 2011 ▸) and one on the structure of 4-phenyl-2-styryl­quinoline (Makela *et al.*, 2021 ▸). In all of these 2-styryl­quinolines, the mol­ecular skeleton is planar, in marked contrast to the nonplanar conformations of the 4-styryl­quinoline derivatives (I)–(III) reported here, and of those reported previously (Rodríguez *et al.*, 2020 ▸). In both 8-styryl­quinoline and 8-[2-(biphenyl-4-yl)vin­yl]-2-methyl­quinoline, the styryl­quinoline fragment is planar (Sharma *et al.*, 2015 ▸), as found in 2-styryl­quinolines but again in marked contrast to 4-styryl­quinolines. It is not easy to see why 4-sty­ryl­quinolines should adopt nonplanar conformations, while mol­ecules of the 2-styryl and 8-styryl isomers appear consistently to adopt planar forms.

## Supplementary Material

Crystal structure: contains datablock(s) global, I, II, III. DOI: 10.1107/S2053229622008634/ky3221sup1.cif

Structure factors: contains datablock(s) I. DOI: 10.1107/S2053229622008634/ky3221Isup2.hkl

Structure factors: contains datablock(s) II. DOI: 10.1107/S2053229622008634/ky3221IIsup3.hkl

Structure factors: contains datablock(s) III. DOI: 10.1107/S2053229622008634/ky3221IIIsup5.hkl

Supporting information file. DOI: 10.1107/S2053229622008634/ky3221Isup5.cml

Supporting information file. DOI: 10.1107/S2053229622008634/ky3221IIsup6.cml

Supporting information file. DOI: 10.1107/S2053229622008634/ky3221IIIsup7.cml

CCDC references: 2204061, 2204060, 2204059

## Figures and Tables

**Figure 2:**
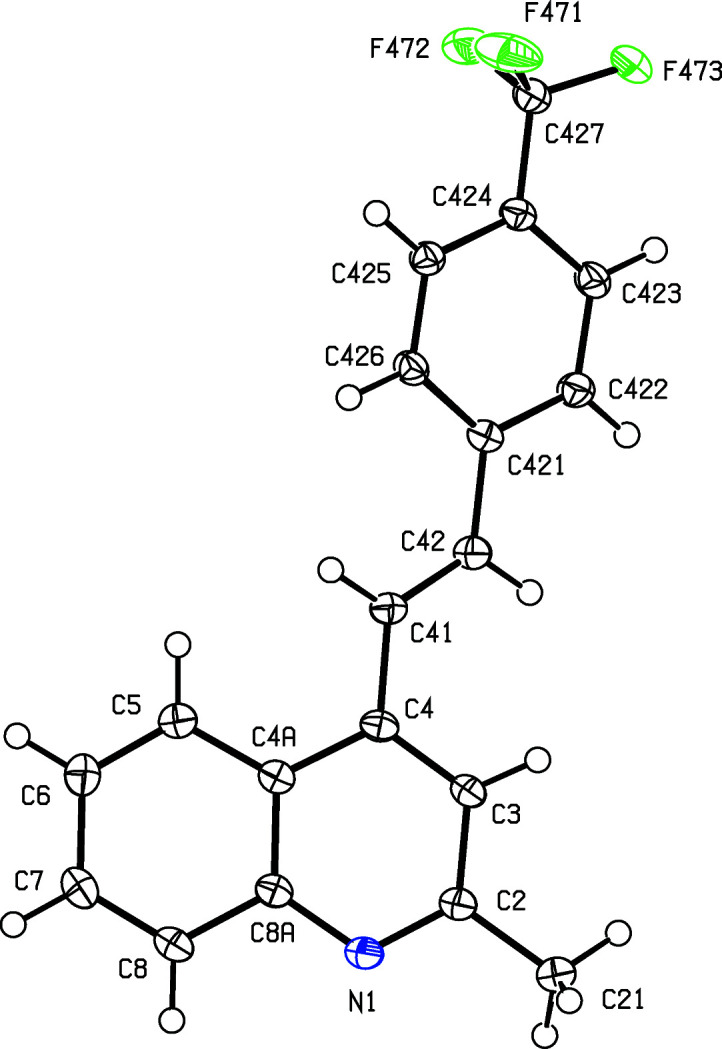
The mol­ecular structure of com­pound (II), showing the atom-labelling scheme. Displacement ellipsoids are drawn at the 50% probability level.

**Figure 3:**
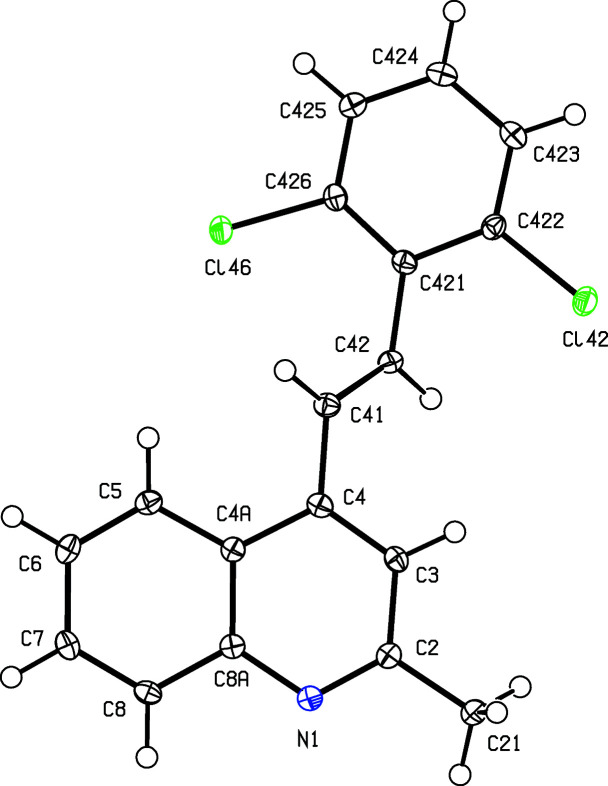
The mol­ecular structure of com­pound (III), showing the atom-labelling scheme. Displacement ellipsoids are drawn at the 50% probability level.

**Table 1:** Experimental details Experiments were carried out at 100 K with Mo *K*α radiation using a Bruker D8 Venture diffractometer. Absorption was corrected for by multi-scan methods (*SADABS*; Bruker, 2016 ▸). H-atom parameters were constrained.

	(I)	(II)	(III)
Crystal data			
Chemical formula	C_18_H_14_FN	C_19_H_14_F_3_N	C_18_H_13_Cl_2_N
*M*_r_	263.30	313.31	314.19
Crystal system, space group	Monoclinic, *P*2_1_/*c*	Monoclinic, *C*2/*c*	Monoclinic, *C*2/*c*
*a*, *b*, *c* (Å)	13.5921 (7), 12.7103 (6), 7.6215 (3)	17.2696 (10), 10.8096 (7), 16.1495 (8)	30.5651 (15), 3.8629 (2), 25.5357 (13)
β (°)	103.133 (2)	91.440 (2)	110.497 (2)
*V* (Å^3^)	1282.25 (10)	3013.8 (3)	2824.1 (2)
*Z*	4	8	8
μ (mm^−1^)	0.09	0.11	0.45
Crystal size (mm)	0.20 × 0.08 × 0.07	0.16 × 0.14 × 0.12	0.20 × 0.10 × 0.06

Data collection			
*T*_min_, *T*_max_	0.934, 0.994	0.888, 0.987	0.897, 0.973
No. of measured, independent and observed [*I* > 2σ(*I*)] reflections	38068, 2949, 2342	46287, 3750, 2921	28110, 3208, 2930
*R*_int_	0.079	0.085	0.042
(sin θ/λ)_max_ (Å^−1^)	0.650	0.667	0.650

Refinement			
*R*[*F* ^2^ > 2σ(*F* ^2^)], *wR*(*F* ^2^), *S*	0.042, 0.100, 1.05	0.047, 0.121, 1.03	0.031, 0.079, 1.07
No. of reflections	2949	3750	3208
No. of parameters	182	209	191
Δρ_max_, Δρ_min_ (e Å^−3^)	0.26, −0.22	0.33, −0.30	0.33, −0.26

Computer programs: *APEX3* (Bruker, 2018 ▸), *SAINT* (Bruker, 2017 ▸), *SHELXT2014* (Sheldrick, 2015*a* ▸), *SHELXL2014* (Sheldrick, 2015*b* ▸) and *PLATON* (Spek, 2020 ▸).

**Table 2:** Selected torsion angles (°) for com­pounds (I)–(III)

Parameter		(I)	(II)	(III)		
C3—C4—C41—C42		38.8 (2)	28.1 (2)	39.5 (2)		
C41—C42—C421—C422		−174.47 (15)	−175.59 (15)	139.58 (15)		

**Table 3:** Parameters (Å, °) for hydrogen bonds and short inter­molecular contacts in com­pounds (I)–(III) *Cg*1 represents the centroid of the N1/C2//C4/C4*A*/C8*A* ring.

	*D*—H⋯*A*		*D*—H	H⋯*A*	*D*⋯*A*	*D*—H⋯*A*
(I)	C8—H8⋯N1^i^		0.95	2.62	3.561 (2)	170
(II)	C7—H7⋯N1^ii^		0.95	2.75	3.678 (3)	168
	C426—H426⋯*Cg*1^iii^		0.95	2.86	3.3627 (17)	114
(III)	C21—H21*A*⋯N1^iv^		0.98	2.63	3.594 (3)	170

Symmetry codes: (i) −*x* + 1, −*y*, −*z* + 1; (ii) −*x* + 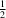 , *y* − 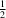 , −*z* + 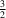 ; (iii) −*x* + 1, −*y* + 1, −*z* + 1; (iv) −*x* + 1, −*y* + 2, −*z* + 1.

## References

[bb1] Alacid, E. & Nájera, C. (2009). *J. Org. Chem.* **74**, 8191–8195. 10.1021/jo901681s 19874064

[bb2] Alkorta, I. & Elguero, J. (1998). *Struct. Chem.* **9**, 59–63.

[bb3] Bernstein, J., Davis, R. E., Shimoni, L. & Chang, N.-L. (1995). *Angew. Chem. Int. Ed. Engl.* **34**, 1555–1573.

[bb4] Bruker (2016). *SADABS*. Bruker AXS Inc., Madison, Wisconsin, USA.

[bb5] Bruker (2017). *SAINT*. Bruker AXS Inc., Madison, Wisconsin, USA.

[bb6] Bruker (2018). *APEX3*. Bruker AXS Inc., Madison, Wisconsin, USA.

[bb7] Cieslik, W., Musiol, R., Nycz, J. E., Jampilek, J., Vejsova, M., Wolff, M., Machura, B. & Polanski, J. (2012). *Bioorg. Med. Chem.* **20**, 6960–6968. 10.1016/j.bmc.2012.10.027 23159041

[bb8] Das, J., Vellakkaran, M. & Banerjee, D. (2019). *Chem. Commun.* **55**, 7530–7533. 10.1039/c9cc03591e 31187810

[bb9] Etter, M. C. (1990). *Acc. Chem. Res.* **23**, 120–126.

[bb10] Etter, M. C., MacDonald, J. C. & Bernstein, J. (1990). *Acta Cryst.* B**46**, 256–262. 10.1107/s0108768189012929 2344397

[bb11] Flack, H. D. & Bernardinelli, G. (1999). *Acta Cryst.* A**55**, 908–915. 10.1107/s0108767399004262 10927300

[bb12] Groom, C. R., Bruno, I. J., Lightfoot, M. P. & Ward, S. C. (2016). *Acta Cryst.* B**72**, 171–179. 10.1107/S2052520616003954 PMC4822653 27048719

[bb13] Gulakova, E. N., Sitin, A. G., Kuz’mina, L. G. & Fedorova, O. A. (2011). *Russ. J. Org. Chem.* **47**, 245–252.

[bb14] Jamal, Z., Teo, Y.-C. & Lim, G. S. (2016). *Tetrahedron*, **72**, 2132–2138.

[bb15] Kamal, A., Rahim, A., Riyaz, S., Poornachandra, Y., Balakrishna, M., Kumar, C., Hussaini, S., Sridhar, B. & Machiraju, P. (2015). *Org. Biomol. Chem.* **13**, 1347–1357. 10.1039/c4ob02277g 25465871

[bb16] Kumar, S., Bawa, S. & Gupta, H. (2009). *Mini Rev. Med. Chem.* **9**, 648–1654. 10.2174/138955709791012247 20088783

[bb17] Kuz’mina, L. G., Sitin, A. G., Gulakova, E. N., Fedorpva, O. A., Lermontova, F. K. & Churakov, A. V. (2011). *Kristollografiya*, **56**, 656–665.

[bb18] Lee, V. M., Gavrishova, T. N. & Budyka, M. F. (2009). *Chem. Heterocycl. C*, **45**, 1279–1280.

[bb19] Leonard, J. T. & Roy, K. (2008). *Eur. J. Med. Chem.* **43**, 81–92. 10.1016/j.ejmech.2007.02.021 17452064

[bb20] Mahajan, S., Gupta, S., Jariwala, N., Bhadane, D., Bhutani, L., Kulkarni, S. & Singh, I. (2018). *Lett. Drug. Des. Discov.* **15**, 937–944.

[bb21] Mäkelä, M. K., Bulatov, E., Malinen, K., Talvitie, J., Nieger, M., Melchionna, M., Lenarda, A., Hu, T., Wirtanen, T. & Helaja, J. (2021). *Adv. Synth. Catal.* **363**, 3775–3782.

[bb22] Meléndez, A., Plata, E., Rodríguez, D., Ardila, D., Guerrero, S. A., Acosta, L. M., Cobo, J., Nogueras, M. & Palma, A. (2020). *Synthesis*, **52**, 1804–1822.

[bb23] Moss, G. P. (1996). *Pure Appl. Chem.* **68**, 2193–2222.

[bb24] Mousnier, A., Leh, H., Mouscadet, J.-F. & Dargemont, C. (2004). *Mol. Pharmacol.* **66**, 783–788. 10.1124/mol.104.001735 15247318

[bb25] Mrozek-Wilczkiewicz, A., Kuczak, M., Malarz, K., Cieślik, W., Spaczyńska, E. & Musiol, R. (2019). *Eur. J. Med. Chem.* **177**, 338–349. 10.1016/j.ejmech.2019.05.061 31158748

[bb26] Mrozek-Wilczkiewicz, A., Spaczynska, E., Malarz, K., Cieslik, W., Rams-Baron, M., Kryštof, V. & Musiol, R. (2015). *PLoS One*, **10**, e0142678. 10.1371/journal.pone.0142678 PMC4657899 26599982

[bb27] Musiol, R. (2020). *Med. Chem.* **16**, 141–154. 10.2174/1573406415666190603103012 31161997

[bb28] Naylor, R. E. & Wilson, E. B. (1957). *J. Chem. Phys.* **26**, 1057–1060.

[bb29] Omar, W. A. E. & Hormi, O. E. O. (2009). *Tetrahedron*, **65**, 4422–4428.

[bb30] Riddell, F. G. & Rogerson, M. (1996). *J. Chem. Soc. Perkin Trans. 2*, pp. 493–504.

[bb31] Riddell, F. G. & Rogerson, M. (1997). *J. Chem. Soc. Perkin Trans. 2*, pp. 249–256.

[bb32] Rodríguez, D., Guerrero, S. A., Palma, A., Cobo, J. & Glidewell, C. (2020). *Acta Cryst.* C**76**, 883–890. 10.1107/S2053229620010803 PMC7474186 32887859

[bb33] Rowland, R. S. & Taylor, R. (1996). *J. Phys. Chem.* **100**, 7384–7391.

[bb34] Sharma, R., Kumar, R., Kumar, L. & Sharma, U. (2015). *Eur. J. Org. Chem.* **2015**, 7519–7528.

[bb35] Sharma, V., Slathia, N., Mahajan, S., Kapoor, K. K. & Gupta, V. K. (2021). *Polyclclic Aromatic Compounds*, https://doi.org/10.1080/10406638.2021.1996409.

[bb36] Sheldrick, G. M. (2015*a*). *Acta Cryst.* A**71**, 3–8.

[bb37] Sheldrick, G. M. (2015*b*). *Acta Cryst.* C**71**, 3–8.

[bb38] Spek, A. L. (2020). *Acta Cryst.* E**76**, 1–11. 10.1107/S2056989019016244 PMC6944088 31921444

[bb39] Tannenbaum, E., Myers, R. J. & Gwinn, W. D. (1956). *J. Chem. Phys.* **25**, 42–47.

[bb40] Valle, G., Busetti, V. & Galiazzo, G. (1986). *Z. Kristallogr. Cryst. Mater.* **177**, 315–318.

